# Design and Performance Evaluation of an Electro-Hydraulic Camless Engine Valve Actuator for Future Vehicle Applications

**DOI:** 10.3390/s17122940

**Published:** 2017-12-18

**Authors:** Kanghyun Nam, Kwanghyun Cho, Sang-Shin Park, Seibum B. Choi

**Affiliations:** 1 School of Mechanical Engineering, Yeungnam University, 280 Daehak-ro, Gyeongsan 712-749, Korea; khnam@yu.ac.kr (K.N.); pss@ynu.ac.kr (S.-S.P.); 2 Samsung Electronics Co., Ltd., Gyeonggi-do 443-742, Korea; khcho0960@gmail.com; 3 Department of Mechanical Engineering, Korea Advanced Institute of Science and Technology, Daejeon 305-701, Korea

**Keywords:** camless engine, electro-hydraulic actuator, snubber design

## Abstract

This paper details the new design and dynamic simulation of an electro-hydraulic camless engine valve actuator (EH-CEVA) and experimental verification with lift position sensors. In general, camless engine technologies have been known for improving fuel efficiency, enhancing power output, and reducing emissions of internal combustion engines. Electro-hydraulic valve actuators are used to eliminate the camshaft of an existing internal combustion engines and used to control the valve timing and valve duration independently. This paper presents novel electro-hydraulic actuator design, dynamic simulations, and analysis based on design specifications required to satisfy the operation performances. An EH-CEVA has initially been designed and modeled by means of a powerful hydraulic simulation software, AMESim, which is useful for the dynamic simulations and analysis of hydraulic systems. Fundamental functions and performances of the EH-CEVA have been validated through comparisons with experimental results obtained in a prototype test bench.

## 1. Introduction

The implementation of a fully variable intake and exhaust valve timing control in an internal combustion engine can significantly improve the fuel efficiency, emissions, and power output [1,2]. The ability to fully vary engine valve timing provides significant improvements to the next generation internal combustion engines. Among variable valve actuation systems, fully flexible systems such as camless actuating equipment is the most attractive valve-train systems for near future engines. A camless engine that enables fully variable valve timing control is able to provide increased power, increased fuel efficiency, and overall reduced emissions at the same time [3,4]. For example, when entering a busy expressway, an engine valve timing controller changes the overlap between intake and exhaust valves for greater engine power. When the vehicle is cruising on the expressway at a constant speed, the timing controller alters the valve timing again to reduce power consumption and increase fuel efficiency. Furthermore, the valve timing can be optimized to reduce emissions. Fuel efficiency can also be increased by deactivating cylinders that do not need operation. When a vehicle is cruising at a low and constant speed, it does not require all cylinders to be operational. A significant amount of research has been conducted to demonstrate the advantage of variable valve actuation for camless engines over the traditional cam-based valve train of both gasoline and diesel engines [5,6,7]. Variable valve actuation (i.e., VVA) or variable valve timing (i.e., VVT) and variable valve lift (i.e., VVL) can be achieved with both electro-mechanical actuators and electro-hydraulic actuators. In [7], new design of camless engines with pneumatic actuator was proposed and its performance was validated from various test results at different engine speeds. Also, an extensive research on the sensors has been carried out to identify the crankshaft position. 

In addition to research on system design, research on control techniques for application to camless engines has been conducted [8,9,10,11,12,13,14,15]. In [8], motion control methods of a camless engine valve actuation system during both steady state and transient engine operation are proposed. Also, the experimental results from a prototype camless engine valve actuation system are then presented to help in validating the overall effectiveness of the proposed control method [10,12] have proposed novel adaptive control strategies to minimize the regulation energy of a new generation of actuators for intake valves of camless engines. Since it consists of a relatively complicated cascade structure including feed-forward actions with an external resonance controller strategy, sufficient amount of computation power should be secured in real-time implementation. A model-based predictive control method has been developed for exhaust electro-pneumatic valve actuators to overcome variable in-cylinder pressure force [11]. Control study for tracking time-varying reference signals and its application to an electro-hydraulic/electro-mechanical type camless engine valve actuator has been done [13,14,15]. In general, the reference engine valve motion is time-varying since its frequency contents change with the engine speed. To achieve the precise time-varying motion control of a nonlinear system, the high-order time-varying internal model is designed for improving the tracking performances [13].

In a field of camless engine technologies, it is very important that the price competitiveness is secured for applications to the actual production vehicle. From this point of view, the sensorless control is one of the most important issues when implementing camless engine technology. Literatures [16,17,18] have proposed new types of observers comprising an augmented extended Kalman filter (EKF) and another EKF as well as nonlinear high gain observers, which results in a cost-effective sensorless control. In literature [19], in order to make the electromagnetic valve train used on exhaust system better, quantitative analysis is carried out against the additional power consumption caused by gas pressure under different conditions. The quantitative analysis aims at making a better compromise between the engine power output and exhaust valves’ power consumption.

In this paper, an electro-hydraulic actuator type was chosen for generating driving force required to make valves open and close due to the advantages in cost-effectiveness and power density. The developed EH-CEVA systems that use pressurized oil as an operating source provide infinitely variable valve timing, duration and lift through independent actuator controls. 

The ultimate objective of this study is to examine the feasibility of an EH-CEVA as a substitute for existing cam-based valve-trains. In order to meet the performance specifications, dynamic simulations and experiments are performed under various operating conditions, and design parameters and control concepts are obtained accordingly.

- EH-CEVA’s dynamic characteristic sensitive to oil temperature, and
- Noise and vibration during valve close

Proposal of mechanical and electrical combined approaches to overcome above challenging issues is one of the contributions of this research. To overcome the first challenge, authors’ research team has proposed real-time control methods that are robust against oil temperature variations [20]. In general, a particularly challenging control obstacle, shown in hydraulic actuation system for camless engine valve actuators, is the sluggish response of valve actuators at a cold operating condition. This is mainly due to the characteristics of oil viscosity with respect to temperature changes. At a low temperature condition, an EH-CEVA shows very sluggish response. The retarded valve opening and closing timing by the EH-CEVA’s slow response at low temperature, may cause an increase in pollutant emission and cylinder temperature during engine operation. In order to avoid these adverse effects by retarded timing, the new valve timing controller is proposed to control opening timing and closing timing, which is robust against temperature variations.

To overcome the second challenge, a novel hydraulic snubber for soft valve landing is designed and parameters affecting operating performance are decided through a simulation study utilizing commercial software, AMESim and MATLAB/Simulink.

Regarding the dynamic model of an EH-CEVA, the authors have built a differential equation governing the dynamics of the EH-CEVA based on mass flow and orifice equations in previous literature [20]. The applied oil pressure in each cylinder is calculated from valve command and supply hydraulic pressure instead of using the non-linear orifice equations [21]. A non-linear hydraulic model considering oil flow dynamics requires much computational time and thereby it is difficult to be implemented in a real time controller. Hence, a linear model with oil temperature and snubber orifice dependent parameter *B* (temperature (*T*), orifice diameter (*D*)) as following equation,

(1) $$M\ddot{x}+B\left(T,D\right)\dot{x}+Kx=F_{\mathrm{actuator}},$$

where, *M* is the mass of an engine valve, *B* is the damping coefficient that is a function of the oil temperature and orifice diameter of a hydraulic snubber, *K* is the spring constant, *x* is the valve displacement, and $F_{\mathrm{actuator}}$ is the actuating force generated by the EH-CEVA [20].

This paper focuses on system design and dynamic simulation using commercial software AMESim. The contribution of this paper is summarized as follows.

- A prototype hydraulic actuator was developed for application to camless engines.
- This paper defined the main design parameters significantly affecting the operating characteristics of EH-CEVA and developed the AMESim simulation model to finally determine the design parameter values.
- To ensure durability of EH-CEVA, a hydraulic snubber design was proposed to minimize the impact force when the valve is closing, and its effects were verified through simulation study based on AMESim and experiments on the test bed.

This paper is composed as follows. In Section 2, the mechanical design of an EH-CEVA is proposed to realize the hydraulic snubber for absorbing the mechanical shock during valve landing. Also, dynamic simulations using the commercial software, AMESim, have been performed to find proper design parametric values. In Section 3, the control system of an EH-CEVA is described and experimental results are presented. In Section 4, conclusions of this paper are described.

## 2. Design and Dynamic Simulation of an EH-CEVA

### 2.1. Mechanical System Design

Design parameters and system specifications required to meet the performance are largely determined by operating conditions of internal combustion engines. At high engine speed, fast valve opening and closing are required with soft valve landing. Specifications of the EH-CEVA are illustrated in Table 1. 

**Figure 1:**
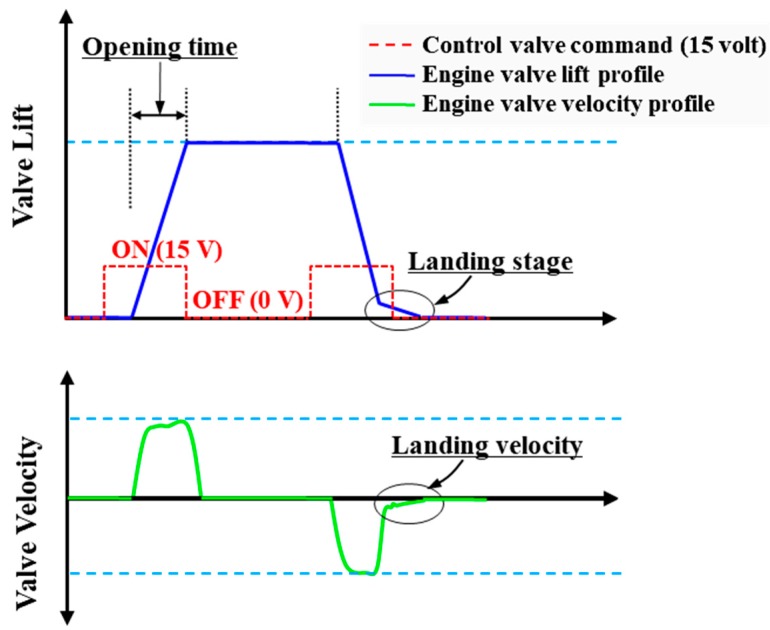
Scheme of the engine valve’s open and close operation.

In order to apply the proposed EH-CEVA system to commercial internal combustion engines, ensuring reliability and endurance is significantly important. Therefore, we proposed a hydraulic snubber design for reducing impact force during valve landing without costly control efforts. As illustrated in Figure 1, the soft valve landing without bouncing is considered a key design requirement. Thus, valve’s landing velocity is an important design specification directly related to soft valve landing. 

**Figure 2:**
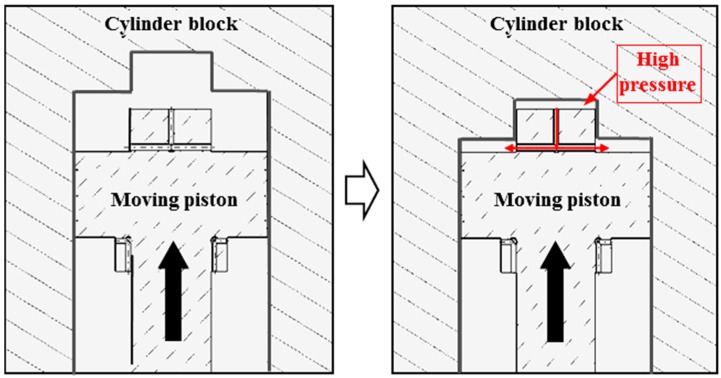
Principle of operation of hydraulic snubber in engine valve closing.

Figure 2, the operation principle of the hydraulic snubber is as follows. In order for the valve to close, high pressure fluid flows into the chamber at the bottom of the piston, and the upper chamber of the piston is connected to the low pressure tank. Then, a force is generated in the upward direction by the pressure difference between upper and lower piston, and the engine valve is closed quickly by adding this force and the spring return force. Several orifices were intentionally applied to the end of the piston that allows instantaneous high pressures to be created in the upper cylinder immediately before the valve is fully closed. The red curve shown in Figure 2 shows that high pressure fluid flows through the orifice just before the engine valve closes. As the diameter of the orifice increases, the flow of fluid through the orifice increases, thus reducing the cushioning effect of the hydraulic snubber. On the other hand, if the orifice diameter decreases, the engine valve will settle smoothly because it takes a long time for the high pressure fluid to completely drain out.

**Figure 3:**
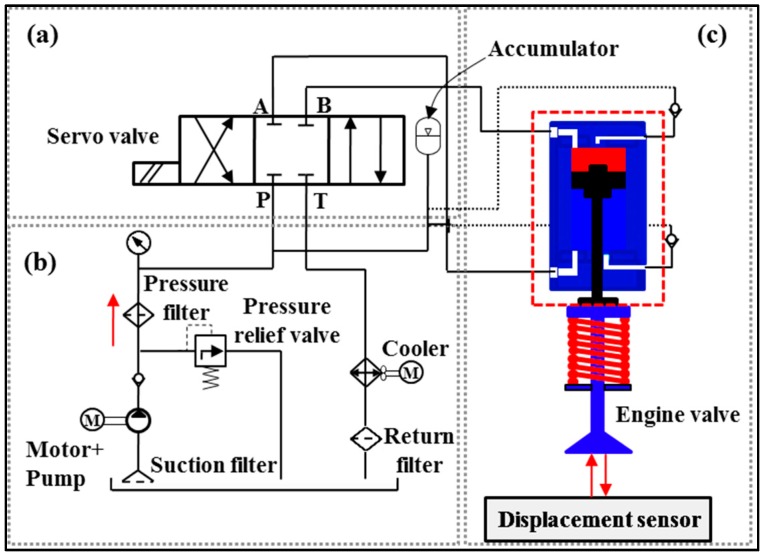
Schematics of the electro-hydraulic camless engine valve actuator (EH-CEVA): (**a**) proportional hydraulic valve module; (**b**) hydraulic power pack module; (**c**) EH-CEVA module.

The schematics of the EH-CEVA is shown in Figure 3. An overall system consists of three modules: a proportional valve control module that is to control the oil flow direction, hydraulic power pack that is to supply an EH-CEVA with actuating force, and a proposed EH-CEVA. 

**Figure 4:**
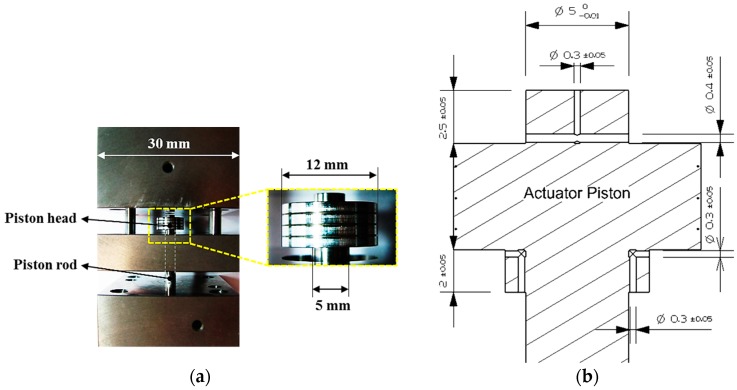
Details of an EH-CEVA: (**a**) proportional hydraulic valve module; (**b**) hydraulic power pack module.

An EH-CEVA consists of two components. One is an actuating piston that induces a linear motion from pressurized operating oil. The other is a cylinder block composed of an upper cylinder block, a middle guide block and a lower cylinder block. The piston, shown in Figure 4a,b, has the orifice control type of a hydraulic snubber that highly contributes to soft valve landing. Actual dimensions for the snubber design suggested in this paper are determined through computer simulations using AMESim under various operating conditions. The hydraulic snubber effects on an EH-CEVA system can be defined as a function of orifice sizes and oil temperature. Therefore, a snubber performance function is expressed as, Snubber performance = *f* (orifice size, oil temperature) [19]. The snubber performance over the specified orifice size range was verified by dynamic simulation using AMESim and from experimental results obtained by prototype bench tests. The valve’s landing velocity is verified by the simulation and experimental results, respectively, shown in Section 3.

### 2.2. Dynamic Simulations Through AMESim-MATLAB//Simulink Co-Simulation

**Figure 5:**
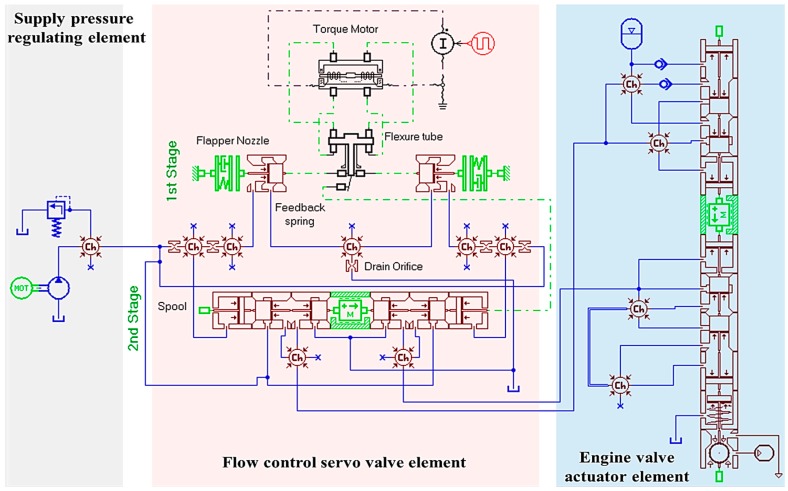
An EH-CEVA AMESim model.

The purpose of this section is to build a simulation model using a hydraulic simulation tool, AMESim. All the hydraulic components and mechanical devices that make the EH-CEVA system have been taken into account in a model. The layout of the AMESim model is shown in Figure 5. In this research, an EH-CEVA AMESim model has been used widely due to a good level of reliability. The EH-CEVA model can be divided into three main elements: (1) supply pressure regulating element, (2) flow control servo valve element, and (3) engine valve actuator element. The supply pressure regulating element consists of an electric motor, a hydraulic pump and a pressure regulator that controls the supplying oil pressure to the servo valve. The flow control servo valve element is a high speed/two stage servo valve. An electric command signal is applied to the motor coils and creates a magnetic force. The force acts on the ends of the pilot stage armature. This causes a deflection of armature/flapper assembly within the flexture tube. Deflection of the flapper restricts oil flow through one nozzle, which is carried through to one spool end displacing the spool. Movement of the spool opens the supply pressure port, i.e., one of the control ports, while simultaneously opening the tank port, i.e., the other control port. The spool position is proportional to the input current. With constant pressure drop across the valve, oil flow to the load is proportional to the spool position. The EH-CEVA creates a hydraulic actuating force that contributes to opening or closing the intake and exhaust engine valves directly.

**Figure 6:**
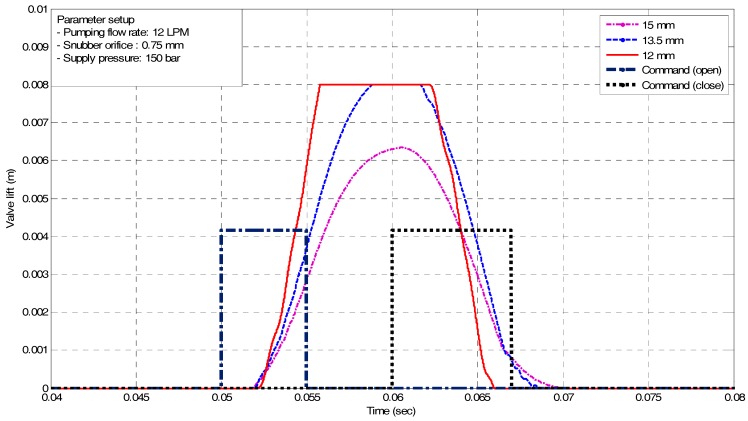
Simulation results for design parameter (e.g., piston diameter) variation.

**Figure 7:**
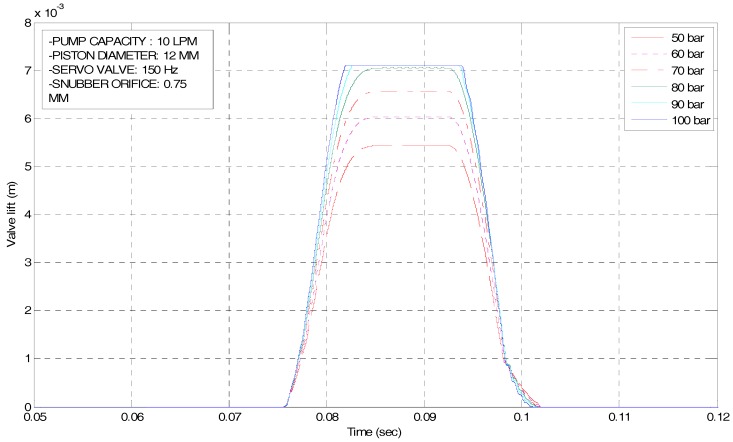
Simulation results for design parameter (e.g., oil supply pressure) variation.

As mentioned, design parameters and system specifications, required to meet the performance, are largely determined by operating conditions of an internal combustion engine. At high engine speed, fast valve opening and closing are needed with soft valve landing. It is noted that key factors influencing the operating characteristics of an EH-CEVA are defined as the supply pressure, flow rate, and piston diameter. The influence of each factor on the operating characteristics of an EH-CEVA was confirmed through simulations using AMESim software. From simulation results, an actuating piston with 12 mm-diameter is selected as a final dimension and the corresponding flow rate from an oil tank is set to 10 LPM (liter per minute). Simulation results for parameter optimization are shown in Figure 6 and Figure 7. An actuating piston diameter and supply pressure, calculated based on performance criteria, are verified through simulations under various parameter conditions. The size selected here represents an optimum design for the defined specifications and known parameters.

## 3. Experimental Verification

### 3.1. Experimental Setup

**Figure 8:**
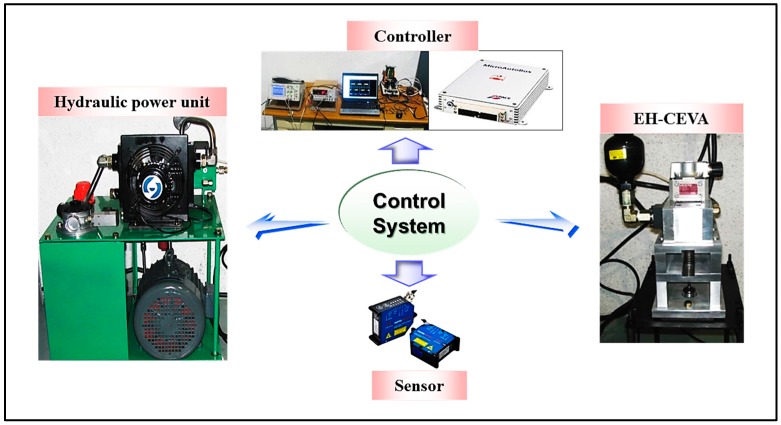
Illustration of the control system.

The real-time control system used in the experiments is illustrated in Figure 8. The control system consists of:

- Hydraulic power unit,
- Developed EH-CEVA,
- Displacement sensor, and
- dSPACE/Micro AutoBox1401 PCI board

An EH-CEVA is installed above an intake valve. A Micro-Epsilon opto-NCDT 1700 CCD laser displacement sensor is used to measure the displacement of an intake valve that is operated by the developed actuator. The laser sensor is mounted on a fixed sensor stage with an angle such that the laser beam from the emitter of the laser sensor is perpendicular to the surface of the end of the valve stem. A Micro Autobox1401 PCI board is used for real-time data acquisition and valve’s open/close timing as well as duration control. DC power supplies are used to provide the electrical power for both the laser sensor and the PCI board.

### 3.2. Experimental Results

The experiments are conducted with open loop control under the combinations of variable system parameters. Experimental results of open loop control under various speeds and experimental results at 2000 rpm are shown in this section. 

**Figure 9:**
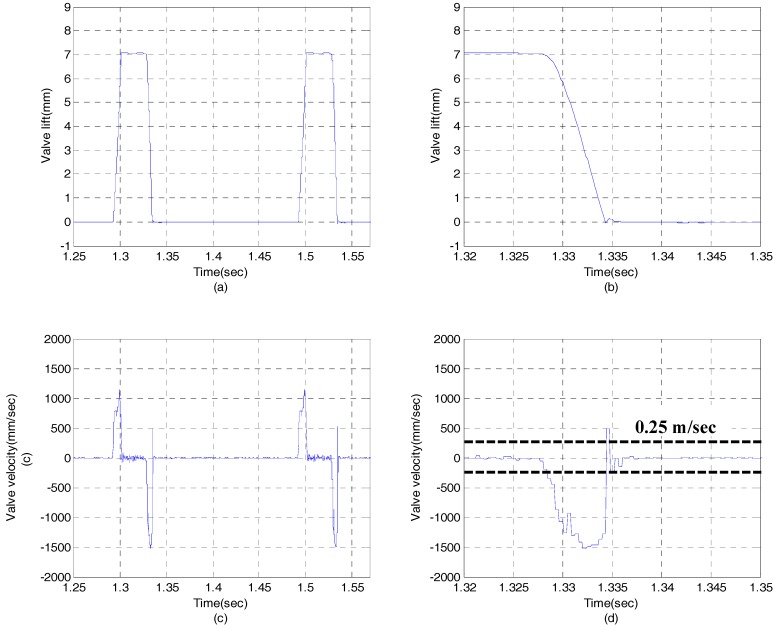
Experimental results for an EH-CEVA I with the 0.8 mm of snubber orifice: (**a**) valve lift profile, (**b**) zoomed in valve lift profile, (**c**) valve velocity profile, (**d**) zoomed-in valve velocity profile.

**Figure 10:**
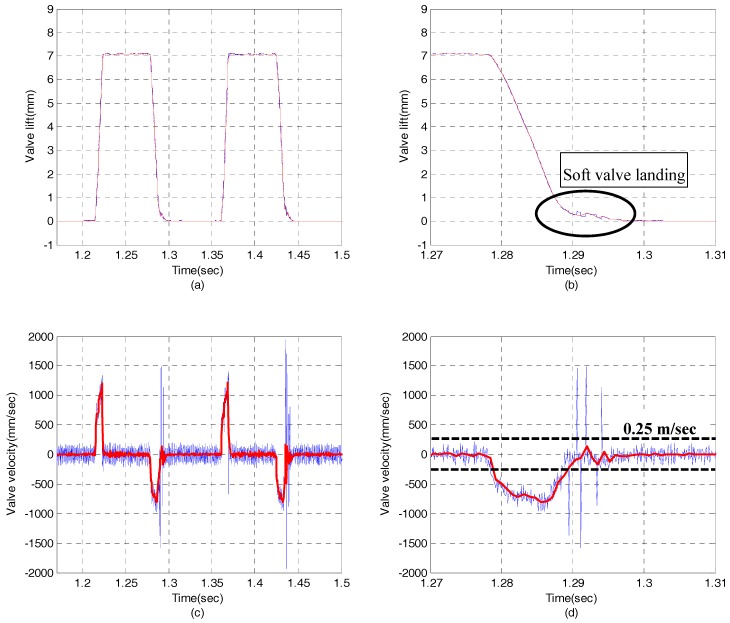
Experimental results for an EH-CEVA with the 0.3 mm of snubber orifice. (blue line: raw measurement data, red line: measurement data with a low-pass filter): (**a**) valve lift profile, (**b**) zoomed in valve lift profile, (**c**) valve velocity profile, (**d**) zoomed-in valve velocity profile.

Figure 9 shows the experimental results in case of EH-CEVA I model with hydraulic snubber. The EH-CEVA I model is initially designed with an orifice size of 0.8 mm. As shown in Figure 9b,d, a hydraulic snubber does not contribute to soft valve landing. On the other hand, Figure 10 shows that the modified actuator model, i.e., EH-CEVA II, meets the performance requirement as expected. The landing velocity of a valve actuator is less than 0.25 m/s which is a target value of valve landing velocity. 

In addition, the response performance of the engine valve can be confirmed through the above experimental results. It should be noted that the valve opening time is sensitive to the supply pressure and the engine speed. Under the current test conditions (i.e., supply pressure = 100 bar and piston diameter = 12 mm), the valve opening time is observed at the level of 5 ms in the low speed region, but it can be observed that the opening time is slightly increased up to 8 ms when the operation speed is increased. It is considered that the response characteristic of the servo valve is deteriorated in the high frequency range and the pressure drop of the servo valve. It is confirmed that test results meets the valve opening time range condition shown in Table 1.

**Figure 11:**
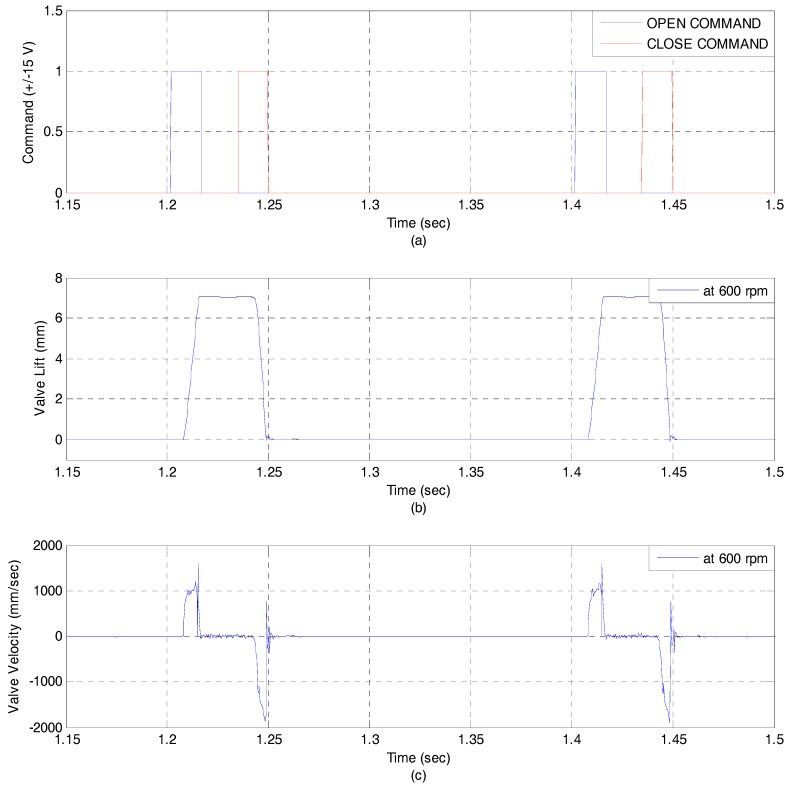
Valve response at 600 rpm and 100 bar (Open loop): (**a**) Open/Close command; (**b**) Valve lift profile; (**c**) Valve velocity profile.

**Figure 12:**
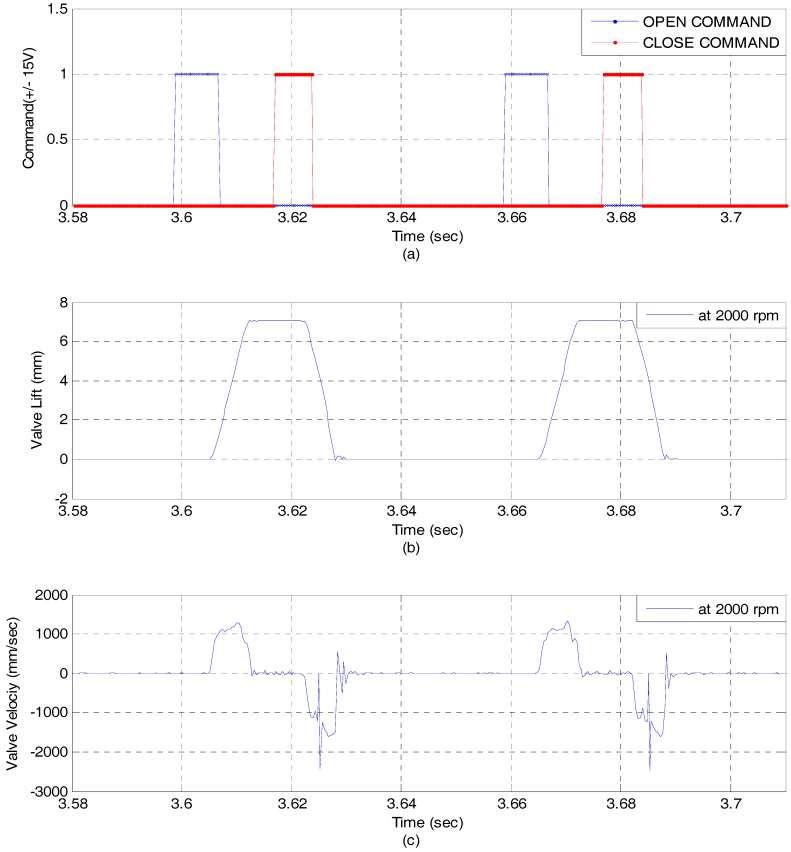
Valve response at 2000 rpm and 100 bar (Open loop): (**a**) Open/Close command; (**b**) Valve lift profile; (**c**) Valve velocity profile.

Figure 11 shows the experimental results at 600 rpm and 100 bar. In order to verify dynamic characteristics, opening time and closing time are measured, respectively. Opening time and closing time are approximately 5 ms. An EH-CEVA is well operated at 600 rpm showing satisfactory opening and closing characteristic. Figure 12 shows the experimental results at 2000 rpm and 100 bar. It is noted that there is not a significant phase lag and delay. An EH-CEVA can be operated up to more than 2000 rpm. 

**Figure 13:**
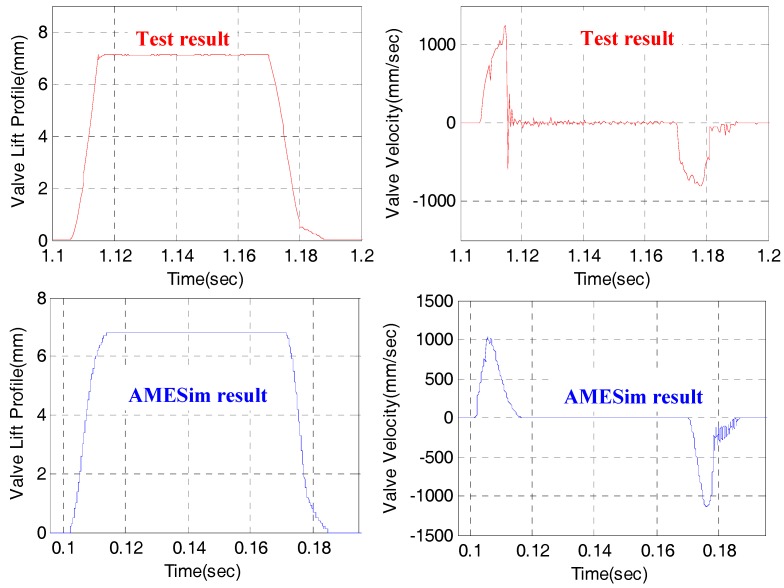
Comparison of valve responses for real EH-CEVA and AMESim model.

In order to evaluate the accuracy of the AMESim model developed in this study compared with the actual EH-CEVA, the engine valve profile results were compared under the same operating conditions. The upper in Figure 13 shows the results obtained from the EH-CEVA experimental test bed, and the lower shows the results obtained from the AMESim model. Although the two profiles of the engine valve are not perfectly matched, this AMESim model can be used to determine the design parameters such as the influence of the hydraulic snubber, the optimal piston diameter, and the supply pressure range, which were studied in this study. In addition, since it is the goal to develop a control system capable of variably controlling the valve timing in accordance with the engine operating conditions, it is expected that it can be usefully used in the control algorithm development.

## 4. Conclusions

In this paper, a new variable valve actuator system, referred to as an EH-CEVA, has been proposed and its characteristics according to design parameter variations has been studied. This paper focuses on the design and performance evaluations of the proposed EH-CEVA system. The system is designed based on numerical analysis and dynamic simulations using a powerful hydraulic analysis software, AMESim. A hydraulic snubber, needed for ensuring endurance and reliability of the EH-CEVA that is applicable to commercial internal combustion engines, is designed and its actual performance and potential are experimentally evaluated on a laboratory test bench. It was confirmed that the developed AMESim model is fairly accurate, and it captures the key characteristics of a hydraulic snubber very well. In future works, new design and manufacturing methods for realizing compactness are proposed and operating performances are also verified through dynamic simulations using the developed AMESim model.

## Figures and Tables

**Table 1:** Design parameters and target specifications.

System Specifications	Target Values
Engine valve lift	7.099 mm
Engine valve diameter	23 mm
Maximum engine speed	4000 RPM
Valve opening time range *	5 ms~10 ms
Valve landing velocity	0.25 m/s
Operating oil temperature	−20 °C to 60 °C

* Valve opening time is defined as the minimum time required for the valve to fully open.
